# Prognostic models for predicting the risk of foot ulcer or amputation in people with type 2 diabetes: a systematic review and external validation study

**DOI:** 10.1007/s00125-021-05448-w

**Published:** 2021-04-27

**Authors:** Joline W. J. Beulens, Josan S. Yauw, Petra J. M. Elders, Talitha Feenstra, Ron Herings, Roderick C. Slieker, Karel G. M. Moons, Giel Nijpels, Amber A. van der Heijden

**Affiliations:** 1 Department of Epidemiology & Data Science, Amsterdam UMC – Location VUmc, Amsterdam Public Health, Amsterdam Cardiovascular Sciences, Amsterdam, the Netherlands; 2 Julius Center for Health Sciences and Primary Care, University Medical Center Utrecht, Utrecht, the Netherlands; 3 Department of General Practice, Amsterdam UMC – Location VUmc, Amsterdam Public Health, Amsterdam, the Netherlands; 4 Groningen Research Institute of Pharmacy, University of Groningen, Groningen, the Netherlands; 5 Centre for Nutrition, Prevention and Health Services, Institute for Public Health and the Environment, Bilthoven, the Netherlands; 6 PHARMO Institute for Drug Outcomes Research, Utrecht, the Netherlands; 7 Department of Cell and Chemical Biology, Leiden University Medical Center, Leiden, the Netherlands

**Keywords:** Amputation, Foot ulcer, Performance, Prognostic model, Systematic review, Type 2 diabetes

## Abstract

**Aims/hypothesis:**

Approximately 25% of people with type 2 diabetes experience a foot ulcer and their risk of amputation is 10–20 times higher than that of people without type 2 diabetes. Prognostic models can aid in targeted monitoring but an overview of their performance is lacking. This study aimed to systematically review prognostic models for the risk of foot ulcer or amputation and quantify their predictive performance in an independent cohort.

**Methods:**

A systematic review identified studies developing prognostic models for foot ulcer or amputation over minimal 1 year follow-up applicable to people with type 2 diabetes. After data extraction and risk of bias assessment (both in duplicate), selected models were externally validated in a prospective cohort with a 5 year follow-up in terms of discrimination (C statistics) and calibration (calibration plots).

**Results:**

We identified 21 studies with 34 models predicting polyneuropathy, foot ulcer or amputation. Eleven models were validated in 7624 participants, of whom 485 developed an ulcer and 70 underwent amputation. The models for foot ulcer showed C statistics (95% CI) ranging from 0.54 (0.54, 0.54) to 0.81 (0.75, 0.86) and models for amputation showed C statistics (95% CI) ranging from 0.63 (0.55, 0.71) to 0.86 (0.78, 0.94). Most models underestimated the ulcer or amputation risk in the highest risk quintiles. Three models performed well to predict a combined endpoint of amputation and foot ulcer (C statistics >0.75).

**Conclusions/interpretation:**

Thirty-four prognostic models for the risk of foot ulcer or amputation were identified. Although the performance of the models varied considerably, three models performed well to predict foot ulcer or amputation and may be applicable to clinical practice.

**Graphical abstract:**

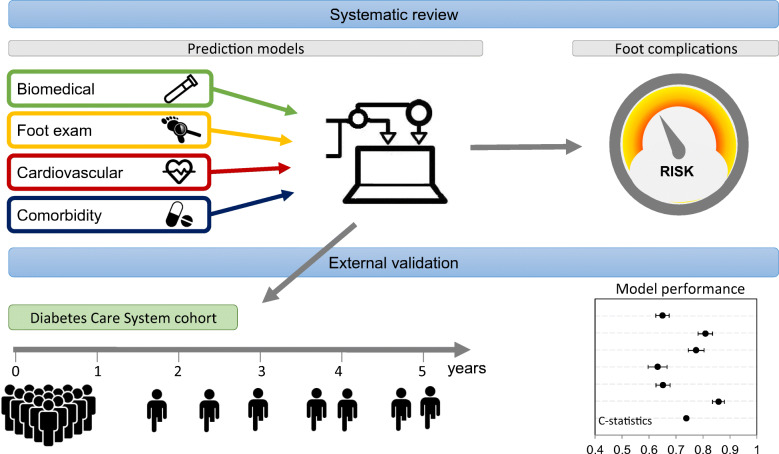

**Supplementary Information:**

The online version contains peer-reviewed but unedited supplementary material available at 10.1007/s00125-021-05448-w.

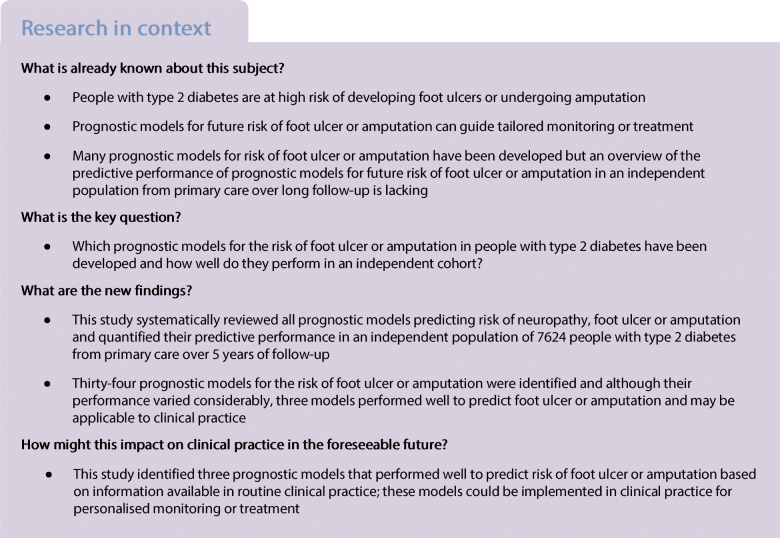



## Introduction

Worldwide, there was an estimated 463 million people living with diabetes in 2019. By 2045 its prevalence is expected to increase to 700 million [1], with 90% being type 2 diabetes. Type 2 diabetes is associated with an increased risk of both macrovascular and microvascular complications [2], including foot ulceration or lower limb amputation. Approximately 15–25% of people with diabetes experience a foot ulcer during their lifetime [3] and people with type 2 diabetes have an approximately ten times higher risk of amputation compared with those without [4]. Therefore, people with type 2 diabetes are monitored annually to assess their risk of foot ulcer and amputation [5].

To guide monitoring frequency or initiate appropriate treatment, the risk of foot ulcer or amputation can be estimated using prognostic models. Such models might be particularly useful in times when prioritisation of routine care is needed, as is the case during the current coronavirus disease 2019 (COVID-19) pandemic. Three main steps should be taken for a prognostic model to be applicable in clinical practice [6]. First, a prognostic model is developed in a prospective cohort or registry. Second, the prognostic model should be validated in an independent population. Third, the impact of the use of the prognostic model in clinical practice on decision making or health outcomes should be tested.

Several prognostic models have been developed to predict the risk of foot ulcer or amputation. A systematic review identified seven risk stratification systems for classifying abnormalities in the foot examination that were developed through a literature review or expert consensus [7]. Another systematic review and meta-analysis developed a prognostic model for foot ulcers among 16,385 people with diabetes [8]. This identified only three predictors: a history of foot ulceration; an inability to feel a 10 g monofilament; and the absence of any pedal pulse [8]. However, the performance of prognostic models for foot ulcer and amputation in an independent population has hardly been investigated. Two small studies of 293 and 446 individuals with diabetes externally validated the risk stratification systems for risk of foot ulcer or amputation identified in a systematic review [7], showing C statistics ranging from 0.56 to 0.86 [9, 10]. However, these systems mainly classified abnormalities in the foot examination and did not incorporate other prognostic factors. Moreover, the majority of the people with diabetes in these validation studies were from a hospital setting with only 223 people from a community-based setting and only included up to 12 months follow-up. These data suggested that the performance of these risk stratification systems was poor in a community-based setting [10]. Therefore, existing prognostic models for the risk of foot ulcer or amputation require external validation particularly in a community-based setting over a longer follow-up period.

The aim of this study was to systematically review all published prognostic models for the risk of foot ulcer or amputation in people with type 2 diabetes, to determine their quality and to quantify their predictive performance in a large community-based independent cohort over 5 years of follow-up.

## Methods

We performed a systematic review and an external validation study. The protocol of the systematic review was registered with the International Prospective Register of Systematic Reviews (PROSPERO) on 21 October 2020 (registration no. CRD42019126838), and the review was performed according to Cochrane guidance for prognostic model reviews [11] and reported according to the PRISMA-P guideline [12]. The external validation study was reported according to the transparent reporting of a multivariable prognostic model for individual prognosis or diagnosis (TRIPOD) guidelines (electronic supplementary material [ESM] Table 1) [13, 14].

### Systematic review

To identify prognostic models for foot ulcer or amputation, we performed a systematic search of PubMed and EMBASE until 21 October 2020. The search term contained several variations of the following keywords: ‘type 2 diabetes’; ‘diabetic foot’ or ‘neuropathy’; and ‘prediction model’ (ESM Table 2 and 3). Studies were included if the following criteria were met: (1) the prognostic model was developed for people with type 2 diabetes or included type 2 diabetes as a predictor; (2) the minimal follow-up period was 1 year; and (3) the outcome was foot ulcer, amputation, neuropathy, or a combination of these. Articles were excluded for the following reasons: non-human studies; studies in languages other than English or Dutch; external validation studies; and if the article was a commentary, review or conference abstract. We included studies with predominantly people with type 2 diabetes or with unspecified diabetes that was suspected to be type 2 diabetes based on individual characteristics. Studies with populations restricted to other forms of diabetes or conducted in populations with predominantly other forms of diabetes were excluded. Titles, abstracts and full texts were screened by two reviewers and by a third reviewer in case of disagreement (JSY, AAH, JWB) using the online tool Covidence (www.covidence.org), which only records inclusion or exclusion of a record. Full text screening was done using an Excel file to record whether a study complied with each inclusion or exclusion criterion to record the main reason for exclusion.

#### Data extraction

Data extraction was conducted by three reviewers (JSY, AAH, JWB) using an Excel file based on the checklist for critical appraisal and data extraction for systematic reviews of prediction modelling studies (CHARMS) [15]. This checklist was developed based on existing reporting guidelines for other types of clinical research and key methodological literature discussing recommended approaches for the design, conduct, analysis and reporting of prediction models. The following domains of CHARMS were used: source of data; participants; outcome(s) to be predicted; candidate predictors; sample size; missing data; model development; model performance; model evaluation; and results.

#### Risk of bias and applicability assessment

Each article was critically appraised for risk of bias and the models’ applicability to the intended population and setting by two reviewers and by a third reviewer in case of disagreement (JSY, AAH, JWB), using an Excel file based on the Prediction study Risk of Bias Assessment Tool (PROBAST) [16, 17]. The risk of bias was assessed for the following domains: the source of data; participants; outcome(s) to be predicted; candidate predictors; missing data; model development; and model performance (ESM Table 4).

### External validation in the Diabetes Care System cohort

To assess the predictive performance of the selected models, we applied the models to the Diabetes Care System (DCS) cohort. The DCS cohort is a large prospective study of people with type 2 diabetes treated in routine primary care from the West-Friesland region of the Netherlands [18]. The DCS cohort is dynamic, with data on risk factors and complications collected annually from 1998 onwards. We used the most recent DCS data from 2014 until 2019 from all participants with sufficient predictor and outcome information available. In 2014, 8348 participants visited our centre and 724 were excluded because of missing data on incidence of foot ulcer or amputation during follow-up, leaving 7624 participants for analysis. We validated all models for the same time period (5 years) to obtain a fair comparison between the models.

The DCS cohort includes several demographic variables (i.e. age, sex), biomedical variables (i.e. systolic BP, HbA_1c_) and variables from foot screening (i.e. monofilament tests) [18]. All predictors were included as a baseline value with 2014 considered baseline. Data on the occurrence and location of ulcers and amputations were retrieved from the medical records from the DCS and the local hospital.

#### Model selection for validation

Retrieved models developed for people with critical limb ischaemia or people with an infected diabetic foot ulcer were excluded from the validation study since such models are not applicable to people with type 2 diabetes treated in primary care. Models were also excluded if important predictors (or proxy variables) were not available in the DCS cohort or if the parameter estimates were not provided in the model development paper and could not be retrieved from the authors.

#### Statistical analyses

To evaluate the predictive performance of the selected models, we used the parameter estimates as stated in each development paper (intercept or baseline hazard and coefficients). If insufficient information was available, the authors were contacted for the original model specification. We validated each model for the outcome for which it was developed and for a combined outcome of 5 year incidence of foot ulcer and amputation.

Differences in the incidence of foot ulcer or amputation in our cohort, and in the development populations, may lead to significant deviation between observed risk in our cohort and predicted risk estimated by the prognostic model. To reduce this source of miscalibration, we ‘recalibrated’ each prognostic model by adjusting the intercept (for logistic regression models) or the baseline survival function (for survival regression models). Each model was validated with and without recalibration of the intercept. For validation without recalibration, we simply applied the model to our data. If the intercept or baseline hazard could not be obtained from the original study, the model was only validated using the incidence derived from the cohort.

Model performance was assessed based on discrimination and calibration. Discrimination describes the ability of the model to distinguish those at high risk of developing foot ulcer or amputation from those at low risk. Discrimination was evaluated using Harrell’s C statistic. Calibration indicates the ability of the model to correctly estimate the absolute risks and was evaluated using calibration plots and the observed/expected ratio. Missing data were handled with multiple imputation. We used five imputation sets and pooled the model performance measures (C statistic and observed/expected ratio) according to Rubin’s rule. All statistical analyses were conducted using 5 R (version 3.6.1) (R Core Team, Vienna, Austria, www.r-project.org) [19] in combination with the following R packages: *mice* (version 3.7.0); *rms* (version 5.1-4); *survival* (version 3.1.8); and *survAUC* (version 1.0.5).

## Results

### Identification of prognostic models for foot ulcer or amputation

The systematic review identified 6933 articles. Of these, the full texts of 203 articles were screened and 21 articles met our inclusion criteria (ESM Fig. 1). The main reasons for exclusion were that articles did not present a prognostic model or used a follow-up shorter than one year.

#### Characteristics of the models

**Fig. 1:**
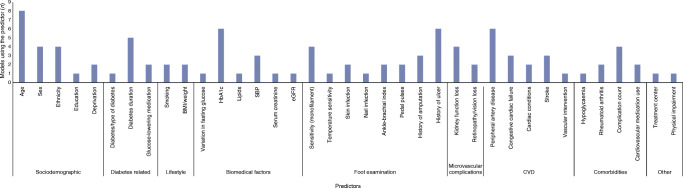
Predictors included in the externally validated prediction models for foot ulcer or amputation. SBP, systolic BP

We identified 21 studies that presented 34 prognostic models to predict the risk of foot ulcer or amputation (Table 1). Most of the studies originated from Europe (*n* = 11) [20–30], followed in frequency by the USA (*n* = 6) [31–36]. One study originated from Japan [37], one study from India [38], one from Taiwan [39] and one study included data from multiple other studies conducted worldwide [40]. Most studies used a study population with diabetes (*n* = 17) and the remaining four studies included diabetes or treatment of diabetes as predictor. Most of the models were developed to predict the risk of amputation (*n* = 16), seven predicted foot ulcer and six predicted some form of diabetic polyneuropathy. The most commonly used prediction horizons were 1 year and 10 years. The number of events ranged from 23 to 3281. The number of predictors included in the 27 models ranged from 2 to 13. An overview of the included predictors is provided in ESM Fig. 2. The most commonly used predictors in the externally validated models were age (*n* = 8), HbA_1c_ (*n* = 6), history of foot ulcer (*n* = 6) or peripheral artery disease (*n* = 6) (Fig. 1).

**Table 1:** Description of the 19 studies identified in the systematic review

Study	Region	Population	Outcome	Sample size	Events (*n*)	Predictors (*n*)	Prediction horizon	Model
Boyko et al, 2006 [31]	USA	95% T2D	Foot ulcer	1285	216	7	1 and 5 years	Final model
Brizuela Sanz et al, 2016 [20]	Europe	CLI >50% diabetes	Death and/or major amputation	561	204	11	1 year	Final model Simplified scale
PODUS, 2015 [40]	Worldwide	T1D & T2D	Foot ulcer	16,385	1221	5	Unclear	Final model
Crawford et al, 2011 [21]	Europe	Unspecified diabetes	Foot ulcer	1192	23	5	1 year	Final model
Dyck et al, 1999 [32]	USA	T1D & T2D	Severity of polyneuropathy	264	NA	4	10 years	Final model
Goodney et al, 2010 [33]	USA	CLI 53% diabetes	Graft occlusion and/or amputation	2036	392	8	1 year	Final model
Hippisley-Cox and Coupland, 2015 [22]	Europe	95% T2D (women)	Lower limb amputation	199,679	1541	11	10 years	Final model
		95% T2D (men)	Lower limb amputation	254,896	3281	13	10 years	Final model
Hurley et al, 2013 [23]	Europe	90% T2D	Foot ulcer	383	19	4	1.5 years	Final model
Iida et al, 2012 [37]	Japan	CLI 69% diabetes	Major amputation	406	69	4	3 years	Final model
Jones et al, 1995 [24]	Europe	Unspecified diabetes	Foot ulcer / amputation	5153	170 / 49	3	Unclear	Final model
Martins-Mendes et al, 2014 [25]	Europe	98% T2D	Foot ulcer	644	171	4	3 years	Final model
						2	3 years	Simplified model
			Amputation	644	37	3	3 years	Final model
						2	3 years	Simplified model
Pickwell et al, 2015 [26]	Europe	Unspecified diabetes	Any amputation	575	159	4	1 year	Risk score
			Amputation excluding lesser toes	575	103	5	1 year	Risk score
Resnick et al, 2004 [34]	USA	Unspecified diabetes	Lower extremity amputation	1974	87	10	8 years	Final model
Tseng et al, 2005 [35]	USA	Unspecified diabetes	Amputation	218,528	3077	3	2 years	Demographic model
						12	2 years	Final model
Venermo et al, 2011 [27]	Europe	CLI Unspecified diabetes	Major lower limb amputation	597	NR	3	1 year	Decision tree
			Amputation free survival	596	NR	3	1 year	Decision tree
Basu et al, 2017 [36]	USA and Canada	T2D	Neuropathy – MNSI >2	9635	3221	12	10 years	Final model
			Neuropathy – vibratory sensation loss	9635	2034	12	10 years	Final model
			Neuropathy – ankle jerk loss	9635	3135	12	10 years	Final model
			Neuropathy – pressure sensation loss	9635	1201	11	10 years	Final model
Dagliati et al, 2018 [28]	Europe	T2D	Neuropathy	943	127	4	3, 5 and 7 years	Nomogram
Beaney et al, 2016 [29]	UK	81% T2D, 19% T1D	Amputation	165	33	7	1 year	Final model Nomogram
Kasbekar et al, 2017 [38]	India	Unspecified diabetes	Amputation	301	83	2	Unclear	Decision tree
Li et al, 2020 [39]	Taiwan	T2D	LEA	21,484	335	13	7.4 years	Risk score
Heald et al, 2019 [30]	UK	Unspecified diabetes	Foot ulcer	17,053	1127	5	5 years	Final model

CLI, critical limb ischaemia; LEA, lower extremity amputation; MNSI, Michigan Neuropathy Screening Instrument; T1D, type 1 diabetes; T2D, type 2 diabetes

#### Risk of bias and applicability

In most studies, the data source was considered a low to moderate risk of bias (ESM Figs 3, 4). The domains missing data, model development and model performance were most often rated as high risk of bias. For eight of the studies missing data was rated as unclear risk of bias. Reasons for the high risk of bias in these domains were not reporting or inappropriate handling of missing data, selecting predictors in the model-based univariable selection or not reporting on measures of discrimination and/or calibration for model performance.

#### Apparent model performance

**Fig. 2:**
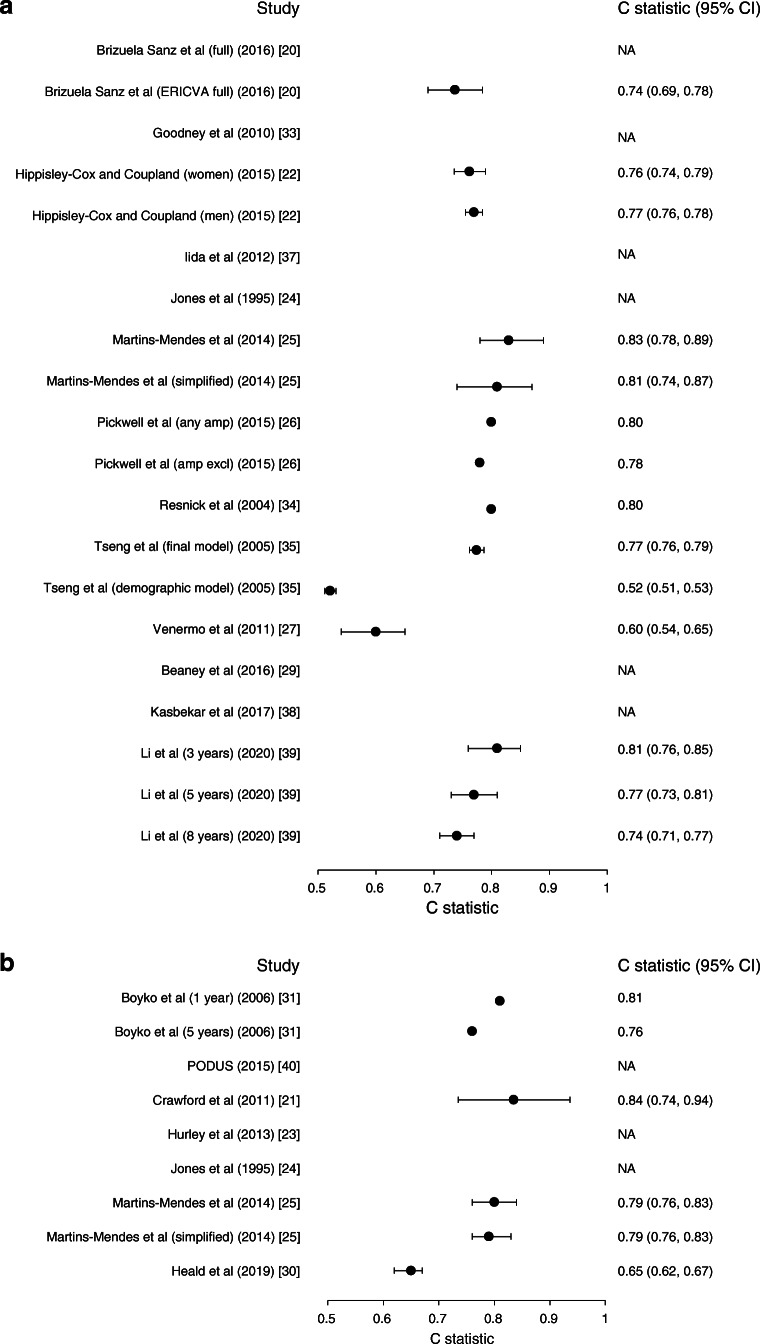
Apparent discrimination of prognostic models for amputation (**a**) and foot ulcer (**b**). amp, amputation; excl, excluded; NA, not applicable because the original study did not report these data

Discrimination was reported in terms of a C statistic for 18 models, of which ten models also presented the 95% CIs. These C statistics (95% CI) ranged from 0.65 (0.62, 0.67) to 0.84 (0.74, 0.94) for models predicting diabetic foot ulcer and from 0.52 (0.51, 0.53) to 0.83 (0.78, 0.89) for models predicting amputation (Fig. 2 and ESM Table 5). For different neuropathy outcomes, C statistics ranged from 0.57 (95% CI 0.55, 0.58) to 0.80 (95% CI not reported). Seven studies reported calibration, which was generally shown to be good. Exceptions to the good calibration were an observed/expected ratio ranging from 0.7 to 1.6 in the study by Goodney et al (2010) [33] and borderline significant calibration tests for models by Venermo et al (*p* ≥ 0.07) and Basu et al (*p* ≥ 0.05) [27, 36].

### External validation

#### Selection of the models

Of the 21 studies reporting on 34 models, 12 were excluded for external validation. The most important reason was that the model was developed for a different target population (*n* = 5) such as people with critical limb ischaemia. Two studies were excluded for external validation because the parameter estimates were not reported and five studies could not be validated because the required predictors or outcome (i.e. incidence of neuropathy) were not available in the cohort (ESM Fig. 1). For some models, predictors were approximated to enable validation. Townsend deprivation score, included in the model of Hippisley-Cox and Coupland [22], was not available in the DCS cohort and the reported sex-specific mean value of the deprivation score was used (men 0.5, women 0.8). Also, the number of cigarettes per day was not recorded in the DCS, and all smokers were assumed to be moderate smokers when applying the model of Hippisley-Cox and Coupland [22]. The model of Martins-Mendes et al [25] used physical impairment as a predictor; this was not registered in the DCS and we assumed that none of the participants were physically impaired. The model of Tseng et al included several specific skin infections [35], and these were assumed absent in all the DCS participants due to unreliable recording of these variables in the DCS.

#### Characteristics of the external validation cohort

Of the 7624 people with type 2 diabetes at baseline, 485 (6.4%) developed a foot ulcer and 70 (0.9%) underwent amputation during the 5 years of follow-up. The mean age of the study population was 67.3 years, 53.1% were male sex and the median duration of diabetes was 7.2 years (Table 2).

**Table 2:** Characteristics of 7624 people with type 2 diabetes from the Hoorn DCS cohort according to the history of ulcer or amputation in 2014

Characteristic	Missing, *n* (%)	Total population	No history of ulcer or amputation	History of ulcer or amputation
*N*		7624	7309	315
Age, years	0	67.3 ± 11.4	67.1 ± 11.4	71.5 ± 11.4
Men	0	4045 (53.1)	3864 (52.9)	181 (57.5)
Education level^a^	67 (0.9)			
Lower, %		3392 (44.5)	3226 (44.1)	166 (52.7)
Medium, %		3079 (40.4)	2968 (40.6)	111 (35.2)
High, %		1153 (15.1)	1115 (15.3)	38 (12.1)
White European ethnicity	18 (0.2)	6971 (91.4)	6666 (91.2)	305 (96.8)
Smoking status	185 (2.4)			
Smoker		1327 (17.4)	1270 (17.4)	57 (18.1)
Former smoker		3747 (49.1)	3598 (49.2)	149 (47.3)
Non-smoker		2550 (33.4)	2441 (33.4)	109 (34.6)
Duration of diabetes, years	1 (0.01)	7.2 (3.5–12.2)	7.1 (3.4–12.1)	10.4 (5.9–15.3)
BMI, kg/m^2^	104 (1.4)	30.2 ± 5.4	30.1 ± 5.4	31.2 ± 6.7
Systolic BP, mmHg	16 (0.2)	141.1 ± 21.0	141.0 ± 21.0	143.5 ± 22.0
Diastolic BP, mmHg	16 (0.2)	78.2 ± 8.4	78.3 ± 8.3	76.6 ± 9.5
Total cholesterol, mmol/l	13 (0.2)	4.5 ± 1.1	4.5 ± 1.1	4.4 ± 1.2
LDL-cholesterol, mmol/l	17 (0.2)	2.4 ± 0.9	2.4 ± 0.9	2.4 ± 1.0
HDL-cholesterol, mmol/l	15 (0.2)	1.3 ± 0.4	1.3 ± 0.4	1.2 ± 0.4
HbA_1c_, mmol/mol	13 (0.2)	51.5 ± 11.9	51.4 ± 11.8	53.9 ± 13.7
HbA_1c_, %	13 (0.2)	6.9 ± 1.1	6.9 ± 1.1	7.1 ± 1.2
Creatinine, μm/l	12 (0.2)	82.8 ± 26.2	82.6 ± 26.1	89.3 ± 28.5
eGFR, ml min^−1^ [1.73 m]^−2^	12 (0.2)	77.3 ± 19.7	77.6 ± 19.7	70.8 ± 20.4
Microalbuminuria	610 (8.0)	1212 (15.9)	1023 (14.0)	189 (60)
Macroalbuminuria	610 (8.0)	98 (1.3)	71 (1.0)	27 (8.6)
Chronic kidney disease	12 (0.2)	105 (1.4)	100 (1.4)	5 (1.6)
Retinopathy^b^	379 (5.0)	396 (5.2)	351 (4.8)	47 (15)
CVD	0	1812 (23.8)	1712 (23.4)	100 (31.7)
CHF	0	158 (2.1)	143 (2.0)	15 (4.8)
Atrial fibrillation	0	287 (3.8)	264 (3.6)	23 (7.3)
Stroke	0	659 (8.6)	629 (8.6)	30 (9.5)
Claudication	0	119 (1.6)	105 (1.4)	14 (4.4)
Rheumatism	0	680 (8.9)	650 (8.9)	30 (9.5)
Glucose-lowering medication use	0			
None		1463 (19.2)	1430 (19.6)	33 (10.5)
Oral		4403 (57.8)	4248 (58.1)	155 (49.2)
Oral and insulin		1353 (17.7)	1264 (17.3)	89 (28.3)
Insulin only		405 (5.3)	367 (5.0)	38 (12.0)
Antihypertensive medication use	0	5555 (72.9)	5292 (72.4)	263 (83.5)
Lipid-lowering medication use	0	5379 (70.6)	5151 (70.5)	228 (72.4)
Foot examination				
Impaired sensibility^c^	238 (3.1)	1856 (24.3)	1639 (22.4)	217 (68.9)
Foot pulses ≤2	422 (5.5)	2858 (37.5)	2665 (36.5)	194 (61.6)
Ulcer 5 year incidence		485 (6.4)	265 (3.6)	220 (69.8)
Amputation 5 year incidence		70 (0.9)	28 (0.4)	42 (13.3)

Data are presented as mean±SD, median (IQR) or *n* (%), unless otherwise stated

^a^Education is defined as low: no education, primary education, secondary education of practical training; medium: prevocational secondary education, vocational training, general secondary education; high: professional university education or university

^b^Retinopathy is defined as grade 1or higher on the Eurodiab grading scale

^c^Impaired sensibility is the inability to perceive monofilament testing

#### Discrimination

**Fig. 3:**
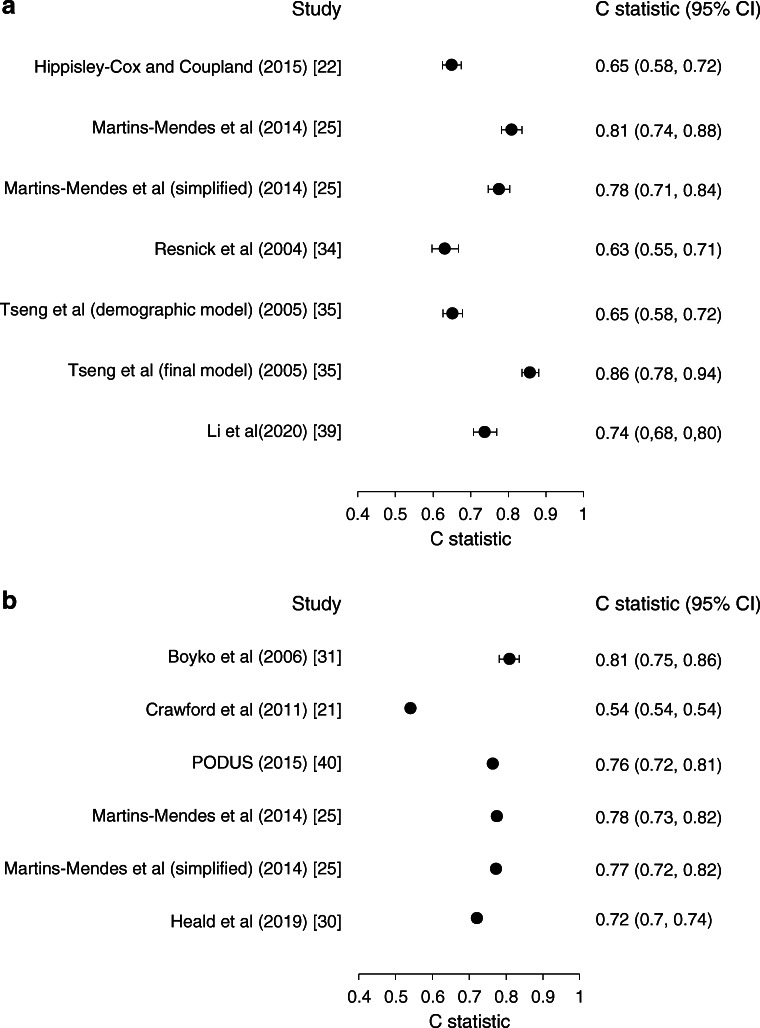
Discriminatory ability of seven prognostic models for amputation (**a**) and six prognostic models for a foot ulcer (**b**) during 5 years in an external validation among 7624 people with type 2 diabetes from the DCS cohort

In the external validation, discriminatory ability of six prognostic models for the development of foot ulcer over 5 years showed C statistics (95% CI) ranging from 0.54 (0.54, 0.54) for the prediction of diabetic foot ulcerations (PODUS) model [40] to 0.81 (0.75, 0.86) for the model by Boyko et al [31] (Fig. 3). For risk of amputation, discriminatory ability of the seven models showed C statistics (95% CI) ranging from 0.63 (0.55, 0.71) for the model by Resnick et al [34] to 0.86 (0.78, 0.94) for the final model by Tseng et al [35] (Fig. 3). For the prediction of the combined outcome of foot ulcer and amputation, C statistics (95% CI) ranged from 0.53 (0.51, 0.55) to 0.84 (0.82, 0.86) (ESM Table 6).

#### Calibration

The calibration plots display the relationship between the predicted risk and observed outcome of the prognostic models after recalibration based on the incidence of the outcome in the DCS cohort (ESM Figs 5–8). In most models, the first quintiles showed agreement between the predicted risk and observed incidence of the particular outcome. Most models generally showed an underestimation of foot ulcer or amputation risk in people in the highest or second-highest risk quintiles. PODUS (2015) [40] and the models by Resnick [34] and Hippisley-Cox and Coupland [22] showed good calibration over all the quintiles.

## Discussion

This systematic review and external validation study provides a comprehensive overview of prognostic models for foot ulcer or amputation and estimated their performance in an independent population. We identified 21 studies that described 34 prognostic models, of which most predicted risk of amputation. Thirteen models could be validated in a large independent community-based population of people with type 2 diabetes. For foot ulcer, most models showed C statistics above 0.75. Performance of models predicting the risk of amputation showed C statistics ranging from 0.63 to 0.86. Predictors included in the models were mostly available in clinical practice and consisted of demographic factors, diabetes-related risk factors, comorbidities and results from the foot examination. Most studies showed a moderate to high risk of bias, mainly due to insufficient reporting on model development.

Most of the identified models predicted future risk of amputation. Of the seven models applicable to the general type 2 diabetes population, three models showed a C statistic around 0.65. However, the other four models showed good discriminatory performance with C statistics over 0.74, which is similar to the performance found in the development populations [25, 35]. Calibration of these models generally showed overprediction of the risk of an ulcer or amputation in the highest predicted risk group. The models by Martins-Mendes et al [25] only contained three predictors (complication count, pulses and previous foot ulcer). In comparison, the model by Tseng et al [35] consisted of 12 predictors including interaction terms using specific information on infections. Therefore, the model by Martins-Mendes [25] seems a very applicable and well-performing model for predicting future risk of amputation. The earlier review by Monteiro-Soares also reported relatively good performance of classification systems for amputation with a C statistic over 0.72, although exact C statistics were not provided [9]. However, this study validated these systems over a prediction horizon of 1 year, which generally results in better performance. This study shows that prognostic models based on foot examination and other characteristics can accurately predict future risk of amputation over a 5 year horizon.

For the prediction of foot ulcers, we were able to validate six prognostic models. These models performed equally well with C statistics around 0.75 for four models, with the exception of the model by Crawford et al (2011) [21]. This model included three predictors, of which two were interaction terms between two variables. Since the model was developed with only 23 events, these predictors may have resulted from overfitting. The other models generally showed performance comparable with the development study, with the exception of PODUS (2015) where discrimination was not reported [40]. The PODUS model (2015) [40] showed good calibration while the other models showed an overprediction of ulcers in the highest risk groups. The five models were based on two to seven predictors and are all relatively easy to apply in clinical practice. Of note, the simplified model by Martins-Mendes was only based on complication count and history of foot ulcer and showed a performance comparable with that of other models [25]. In the study by Monteiro-Soares, risk classification systems based on the foot examination were validated for their prognostic performance to predict future foot ulcers [10]. In a hospital setting, these systems performed well to predict foot ulcers but in a community-based setting discriminatory performance was generally below 0.7. Our study shows that prognostic models including other predictors next to the foot examination perform well to predict future risk of foot ulcers in a community-based setting over a 5 year horizon.

Adequate performance of a model based on calibration and discrimination is a prerequisite for its application in clinical practice. Once this is evaluated, clinical applicability is the leading consideration for choosing a suitable model. With this external validation study, we identified models from four studies that performed sufficiently well to allow such clinical application. For ease of use in clinical practice, the most suitable prognostic model is based on only a few predictors that are readily available in clinical practice. Since we evaluated predictive performance for two closely linked outcomes, foot ulcer and amputation, the prognostic model of choice should preferably perform well to predict both outcomes simultaneously. Using a combined endpoint, only the models of Boyko et al [31], PODUS (2015) [40] and Martins-Mendes et al [25] showed good performance with C statistics of 0.75 or over. These studies also showed low or moderate risk of bias for most domains, except one for PODUS (2015) [40] and two for Martins-Mendes et al [25]. Although the PODUS (2015) model and the model by Martins-Mendes et al contain predictors that are mostly available in clinical practice, the model by Boyko et al also contains predictors requiring further diagnostic testing such as visual acuity or infections of the nails. Such predictors make a model less feasible to implement in clinical practice. Furthermore, a calculation tool such as an Excel spreadsheet, provided by Boyko et al, may enable implementation in routine practice. Of note, all models contain history of foot ulcer or amputation as predictors. Future studies should thus update the models to apply them for primary prevention. Nevertheless, the models identified in this study can be used to guide monitoring strategies in people with type 2 diabetes based on their predicted risk of foot ulcer and amputation. In people at low risk of foot complications, the current routine screening interval can be extended. Such a personalised screening frequency can be established using an optimal balance between the risk of delayed detection of foot complications and the costs of foot screening. This approach was previously used to personalise monitoring frequency for retinopathy based on prediction models [41, 42]. A recent observational study of 10,421 people with diabetes showed that only 5.1% of those classified as low risk had progressed to moderate risk in 2 years [43]. If people with diabetes change risk status infrequently, then regular foot screening is less likely to be of clinical value and personalised screening intervals could be valuable. Personalised monitoring based on prognostic models for foot ulcer and amputation could substantially reduce patient and clinician burden in the management of people with type 2 diabetes. The clinical utility of a prediction model is also dependent on the interventions available for those at high risk. There are a number of effective interventions for preventing foot ulceration and as a result amputations, including the reduction of peak foot pressure, removing excessive callus, accommodating foot deformities [44] and adequate information and education of the individual with type 2 diabetes for foot care [45]. Further research could focus on the development of such a stratification and monitoring system, where the prevalence of false-negative individuals should be foremost in determining monitoring intervals.

A limitation of our study is the low incidence of amputation in the DCS cohort. Validation studies to test model performance require at least 100 events [46, 47]. This criterion was fulfilled for incidence of foot ulcer but the analysis may have been slightly underpowered for incidence of amputation. However, we observed stable estimates for risk of amputation with relatively small CIs, suggesting that the limited number of events did not affect our results to a large extent. Another limitation of our study is the inability to differentiate between major and minor imputations. These data were only available for part of the events and further restricting an already infrequent outcome in our population would result in too few events to draw conclusions. Furthermore, the study population was primarily of European descent and from a centrally organised care centre with standardised protocols. This may limit the extrapolation to populations with a different migration background and people with diabetes in a less-standardised care setting. Although certain models were directly applicable to our data, a final limitation is that not all predictors used by the models were available in the DCS cohort. However, data from the DCS cohort arises from routine clinical practice and the first prerequisite of a model to be used in clinical practice is the availability of the predictors in routinely collected data. This study also has several strengths. First, the prognostic models were identified through a systematic review. Second, the use of a large, unselected cohort of people with type 2 diabetes, including almost all people with type 2 diabetes in the catchment area of the DCS, enhances this external validation study. Third, all predictor and outcome measurements in the cohort were performed according to centrally standardised protocols, which enhanced the reliability of the data and resulted in a low number of missing variables. Finally, the results of the validation study apply to routine clinical practice settings for people with type 2 diabetes.

In conclusion, this systematic review and external validation study identified 34 prognostic models for future risk of foot ulcer or amputation. The external validation of 13 models showed that most models performed well to predict foot ulcer or amputation over a 5 year horizon. The models by Boyko et al [31], PODUS (2015) [40] and Martins-Mendes et al [25] performed best in predicting the combined endpoint of ulcer and amputation, and contain easy-to-measure predictors, making them suitable for clinical practice. These prognostic models could be used to tailor the screening frequency of the foot examination based on individual risk predictions, and may highly increase the efficiency of foot care.

## Supplementary information

ESM (PDF 736 kb)

## Data Availability

Data from this study are available upon request to the steering committee of the Hoorn studies (email: hoornstudies@amsterdamumc.nl).

## Abbreviations

CHARMS: Checklist for critical appraisal and data extraction for systematic reviews of prediction modelling studies
DCS: Diabetes Care System
PODUS: Prediction of diabetic foot ulcerations
