# Supplementary material for: Prognostic models for predicting the risk of foot ulcer or amputation in people with type 2 diabetes: a systematic review and external validation study
Source: Diabetologia. 2021 Apr 27;64(7):1550–62. doi: 10.1007/s00125-021-05448-w (PMC8075833; doi:10.1007/s00125-021-05448-w)

Electronic supplementary material (ESM)

ESM Table 1: TRIPOD-check list for prediction model validation

Section/Topic		Checklist Item	Page
**Title and abstract**			
Title	1	Identify the study as developing and/or validating a multivariable prediction model, the target population, and the outcome to be predicted.	1
Abstract	2	Provide a summary of objectives, study design, setting, participants, sample size, predictors, outcome, statistical analysis, results, and conclusions.	2
**Introduction**			
Background and objectives	3a	Explain the medical context (including whether diagnostic or prognostic) and rationale for developing or validating the multivariable prediction model, including references to existing models.	4
	3b	Specify the objectives, including whether the study describes the development or validation of the model or both.	5
**Methods**			
Source of data	4a	Describe the study design or source of data (e.g., randomized trial, cohort, or registry data), separately for the development and validation data sets, if applicable.	7
	4b	Specify the key study dates, including start of accrual; end of accrual; and, if applicable, end of follow-up.	7
Participants	5a	Specify key elements of the study setting (e.g., primary care, secondary care, general population) including number and location of centres.	7
	5b	Describe eligibility criteria for participants.	7
	5c	Give details of treatments received, if relevant.	NA
Outcome	6a	Clearly define the outcome that is predicted by the prediction model, including how and when assessed.	6,7
	6b	Report any actions to blind assessment of the outcome to be predicted.	NA
Predictors	7a	Clearly define all predictors used in developing or validating the multivariable prediction model, including how and when they were measured.	7, 26
	7b	Report any actions to blind assessment of predictors for the outcome and other predictors.	NA
Sample size	8	Explain how the study size was arrived at.	7
Missing data	9	Describe how missing data were handled (e.g., complete-case analysis, single imputation, multiple imputation) with details of any imputation method.	8, 9
Statistical analysis methods	10c	For validation, describe how the predictions were calculated.	8, 9
	10d	Specify all measures used to assess model performance and, if relevant, to compare multiple models.	8,9
	10e	Describe any model updating (e.g., recalibration) arising from the validation, if done.	8, 9
Risk groups	11	Provide details on how risk groups were created, if done.	NA
Development vs. validation	12	For validation, identify any differences from the development data in setting, eligibility criteria, outcome, and predictors.	8
**Results**			
Participants	13a	Describe the flow of participants through the study, including the number of participants with and without the outcome and, if applicable, a summary of the follow-up time. A diagram may be helpful.	7
	13b	Describe the characteristics of the participants (basic demographics, clinical features, available predictors), including the number of participants with missing data for predictors and outcome.	12
	13c	For validation, show a comparison with the development data of the distribution of important variables (demographics, predictors and outcome).	19-21
Model performance	16	Report performance measures (with CIs) for the prediction model.	12, 23
Model-updating	17	If done, report the results from any model updating (i.e., model specification, model performance).	12, 33, 35
**Discussion**			
Limitations	18	Discuss any limitations of the study (such as nonrepresentative sample, few events per predictor, missing data).	15, 16
Interpretation	19a	For validation, discuss the results with reference to performance in the development data, and any other validation data.	13, 14
	19b	Give an overall interpretation of the results, considering objectives, limitations, results from similar studies, and other relevant evidence.	14, 15
Implications	20	Discuss the potential clinical use of the model and implications for future research.	14, 15

Other information			
Supplementary information	21	Provide information about the availability of supplementary resources, such as study protocol, Web calculator, and data sets.	24,25
Funding	22	Give the source of funding and the role of the funders for the present study.	17

ESM Table 2: Search terms used for systematic review

Database	Number of items identified	Search term
Pubmed	2,555	(("Peripheral Nervous System Diseases"[Mesh] OR neuropath*[tiab] OR amputat*[tiab] OR ulcerat*[tiab]) AND (Validat*[tiab] OR validit*[tiab] OR Predict*[tiab] OR Rule*[tiab] OR (Decision*[tiab] AND (Model*[tiab] OR Clinical[tiab])) OR (Prognostic[tiab] AND (History[tiab] OR Variable*[tiab] OR Criteria[tiab] OR Score[tiab] OR Scores*[tiab] OR Characteristic*[tiab] OR Finding*[tiab] OR Factor*[tiab] OR Model*[tiab])) OR risk score*[tiab] OR risk assessment*[tiab] OR algorithm*[tiab]) AND ("Diabetes Mellitus"[Mesh] OR diabetes[tiab] OR (diabetic*[tiab] AND (non insulin depend*[tiab] OR noninsulin depend*[tiab] OR noninsulindepend*[tiab] OR non insulindepend*[tiab])) OR dm2[tiab] OR niddm[tiab] OR dm 2[tiab] OR t2d*[tiab] OR dm type 2[tiab] OR type 2 diabet*[tiab] OR type two diabet*[tiab] OR type II diabet*[tiab] OR dm type II[tiab])) NOT ("Animals"[Mesh] NOT "Humans"[Mesh])
Embase	4,175	'peripheral neuropathy'/exp OR neuropath*:ab,ti OR amputat*:ab,ti OR ulcerat*:ab,ti<br><br>validat*:ab,ti OR validit*:ab,ti OR predict*:ab,ti OR rule*:ab,ti OR (decision* NEAR/3 (model* OR clinical)):ab,ti OR (prognostic NEAR/3 (history OR variable* OR criteria ORscore OR scores* OR characteristic* OR finding* OR factor* OR model*)):ab,ti OR 'risk score*':ab,ti OR 'risk assessment*':ab,ti OR algorithm*:ab,ti<br><br>'diabetes mellitus'/exp OR diabetes:ab,ti OR (diabetic* NEAR/3 ('non insulin depend*' OR 'noninsulin depend*' OR noninsulindepend* OR 'non insulindepend*')):ab,ti ORdm2:ab,ti OR niddm:ab,ti OR 'dm 2':ab,ti OR t2d*:ab,ti OR 'dm type 2':ab,ti OR 'type 2 diabet*':ab,ti OR 'type two diabet*':ab,ti OR 'type ii diabet*':ab,ti OR 'dm type ii':ab,ti<br><br># NOT ([animals]/lim NOT [humans]/lim)

Both databases together resulted in the identification of 4,588 items (without duplicates).

ESM Table 3: PICOTS items framing the review aim, search strategy, and study inclusion and exclusion criteria for the systematic review

Item	Description
Population	People with type 2 diabetes or applicable to people with type 2 diabetes by including it as predictor
Intervention or Model	All prognostic models to predict risk of foot ulcer and amputation
Comparator	Not applicable
Outcome(s)	Neuropathy, foot ulcer or amputation or a combination of these
Timing	At least 1 year follow-up
Setting	Applicable to people with type 2 diabetes treated in primary care

ESM Table 4: risk of bias assessment rules

Risk of bias domain	Low risk of bias	Moderate risk of bias	High risk of bias
Source of data	Cohort or RCT	Registry	Case-control or cross-sectional
Participants	Appropriate inclusion/exclusion participants	-	Exclusion of specific subgroups
Outcome(s) to be predicted	Clear (pre-specified) definition of the outcome, outcome assessed similarly for all participants	Unclear (no pre-specified) definition of the outcome or outcome assessed differently for all participants, and outcome assessors not blinded from predictor information	Unclear (no pre-specified) definition of the outcome, outcome assessed differently for all participants and outcome assessors not blinded from predictor information
Candidate predictors	Clear definition of the predictors, predictors assessed similarly for all participants, and continuous predictors handled as continuous	Unclear definition of the predictors and/or predictors assessed differently for all participants and/or continuous predictors handled as categorical	Unclear definition of the predictors, predictors assessed differently for all participants and continuous predictors handled as categorical
Missing data	Multiple imputation was used	Single imputation was used	Complete case analysis was used
Model development	Complexities (time-to-event, competing risk, multiple events and multiple centers) were accounted for and variable selection based was not based on univariable analysis	Complexities (time-to-event, competing risk, multiple events and multiple centers) were not accounted for or variable selection based was based on univariable analysis	Complexities (time-to-event, competing risk, multiple events and multiple centers) were not accounted for and variable selection based was based on univariable analysis
Model performance	Discrimination and calibration were assessed	Discrimination or calibration was not assessed	Discrimination and calibration were not assessed

ESM Table 5: summary of apparent model performance measures

Article	Model	Discrimination	Calibration	Other
Boyko 2006	Year 1	C = 0.81	NR	NR
	Year 5	C = 0.76	NR	NR
Brizuela Sanz 2016	Main	NR	NR	NR
	ERICVA scale	Development: 0.737 (0.690, 0.784)<br>Internal validation: 0.708 (0.599, 0.812)	NR	NR
PODUS 2015	Main	NR	NR	NR
Crawford 2011	Main	0.835 (0.735, 0.936)	NR	Sens= 25.0%<br>Spec= 99.3%
Dyck 1999	T1D & T2D	NR	NR	R^2= 0.33
Dyck 1999	T2D	NR	NR	R^2= 0.26
Goodney 2010	Main	NR	O/E ratio= 0.7 - 1.6	NR
Hippisley-Cox	Women	Validation 1= 0.762 (0.735, 0.789)<br>Validation 2= 0.700 (0.670, 0.731)	Plots	Sens= 33.2-59.8%<br>Spec= 80.2-90.2%
	Men	Validation 1= 0.770 (0.755, 0.784)<br>Validation 2= 0.748 (0.730, 0.767)	Plots	Sens=37.5-58.0%<br>Spec=80.4-90.4%
Hurley 2013	Main	NR	NR	Risk stratification in 3 groups
Iida 2012	Main	NR	NR	Risk stratification in 3 groups
Jones 1995	Main	NR	NR	Sens= 72.0 - 95.8%<br>Spec= 59.3 - 84.7%
Martins-Mendes 2014	Ulcer	0.80 (0.76, 0.84)	NR	NR
Martins-Mendes 2014	Ulcer, simplified	0.79 (0.76, 0.83)	NR	NR
Martins-Mendes 2014	Amputation	0.83 (0.78, 0.89)	NR	NR
Martins-Mendes 2014	Amputation, simplified	0.81 (0.74, 0.87)	NR	NR
Pickwell 2015	Any amputation	0.80	NR	NR

Pickwell 2015	Amputation excl. toes	0.78	NR	NR
Resnick 2004	Main	0.80	H-L p=0.88	NR
Tseng 2005	Final model	Development= 0.825<br>Internal validation= 0.774 (0.762, 0.787)	O/E ratio = 0.85 - 1.15	R^2= 0.197<br>R^2= 0.184 (0.171, 0.195)
Tseng 2005	Demographic model	Development= 0.553<br>Internal validation= 0.521 (0.512, 0.531)	O/E ratio = 0.79 - 1.25	R^2= 0.006<br>R^2= 0.005 (0.003, 0.007)
Venermo 2011	Amputation	0.60 (0.54, 0.65)	H-L p=0.31	NR
Venermo 2011	Amputation-free survival	0.65 (0.60, 0.69)	H-L p=0.07	NR
Basu 2017	MNSI>2	0.60 (0.59-0.62)	GDN p=0.11	NR
	Vibratory sensation loss	0.64 (0.63-0.66)	GDN p=0.05	NR
	Ankle jerk loss	0.57 (0.55- 0.58)	GDN p=0.84	NR
	Pressure sensation loss	0.62 (0.61- 0.64)<br>Validation= 0.69 (0.63-0.74)	GDN p=0.37<br>p=0.91	NR
Dagliati 2018	Neuropathy 3 years	0.799	NR	Sens=0.783<br>Spec=0.707
	Neuropathy 5 years	0.714	NR	Sens=0.667<br>Spec=0.697
	Neuropathy 7 years	0.769	NR	Sens=0.688<br>Spec=0.780
Beaney 2016	Amputation	NR	NR	NR
Kasbekar 2017	Amputation	Accuracy=95%	NR	Kappa=0.88
Li 2020	LEA	D: 3-yr: 0.80 (0.76-0.83); 5-yr: 0.78 (0.75–0.81), 8-yr: 0.76 (0.74–0.79)	H-L p>0.05	Sens=83.1%<br>Spec=52.1%
		V: 3-yr: 0.81 (0.76–0.85), 5-yr: 0.77 (0.73–0.81), 8-yr: 0.74 (0.71–0.77)	Calibration plots	
Heald 2019	Foot ulcer	0.65 (0.62-0.67)	Absolute risks in deciles	NR

NR: not reported; C: C-statistic; O/E ratio: observed/expected ratio; Sens: sensitivity; Spec: Specificity; MNSI: Michigan Neuropathy Screening Instrument; GDN: Greenwood-D'Agostino-Nam test; LEA: lower extremity amputation

ESM Table 6. C-statistics for 5-year prediction of a combined outcome of ulcer or amputation for 13 externally validated prognostic models

	C-statistic	Lower CI	Upper CI
Boyko, 2006	0.84	0.82	0.86
Crawford, 2011	0.56	0.54	0.57
PODUS 2015	0.75	0.73	0.77
Martins-Mendes 2014 – for ulcer	0.77	0.75	0.79
Martins-Mendes 2014 simplified for ulcer	0.77	0.75	0.79
Hippisley-Cox 2015	0.61	0.58	0.63
Martins-Mendes 2014 –for amputation	0.77	0.75	0.79
Martins-Mendes 2014 simplified for amputation	0.77	0.75	0.79
Resnick 2004	0.54	0.51	0.56
Tseng 2005 - basic	0.53	0.51	0.55
Tseng 2005	0.59	0.57	0.61
Li 2020	0.74	0.73	0.75
Heald 2019	0.72	0.71	0.73

ESM Figure 1: PRISMA 2009 Flow Diagram

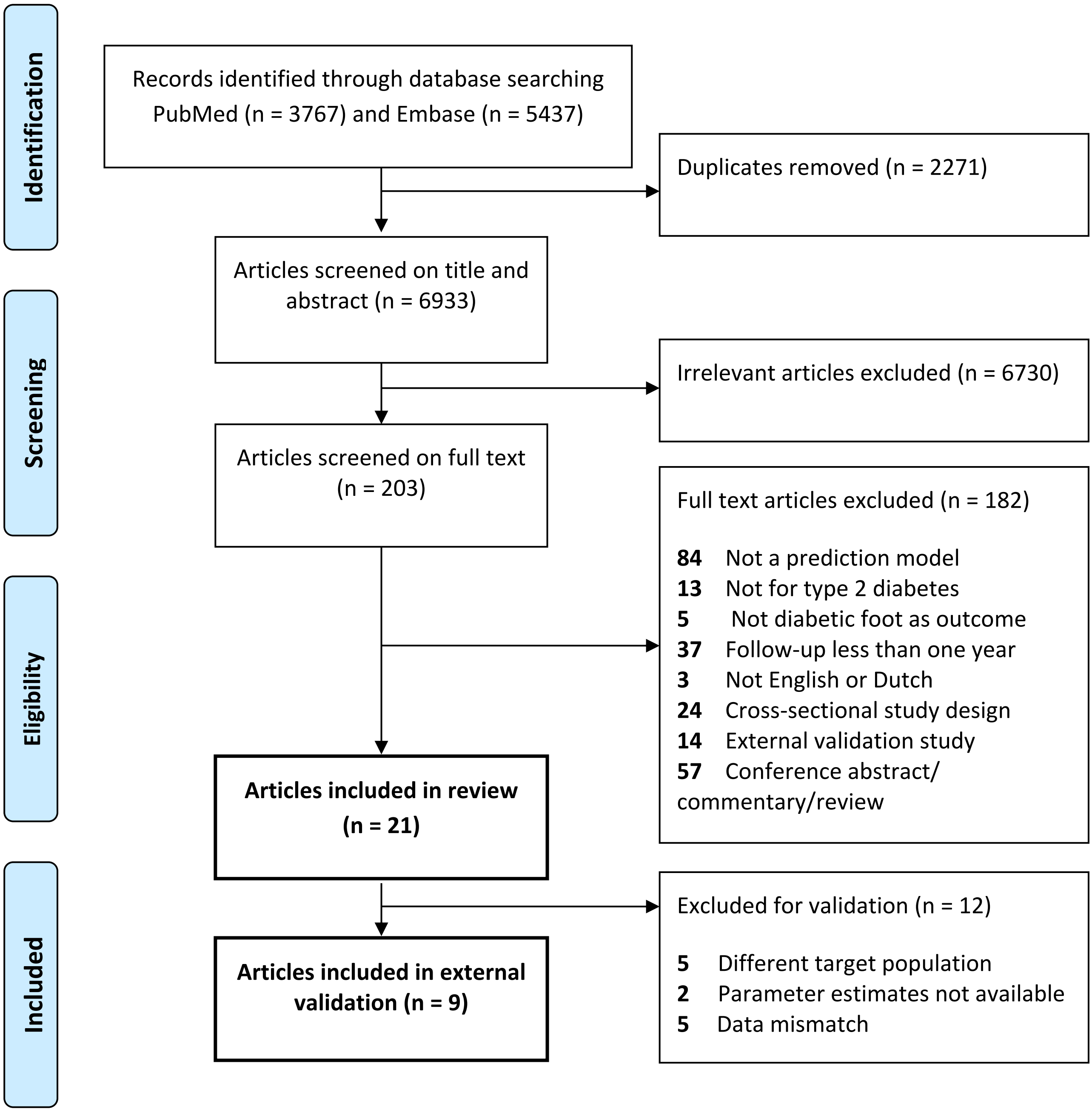

ESM Figure 2: Frequency of predictors included in 21 studies with 34 prognostic models for foot ulcer or amputation

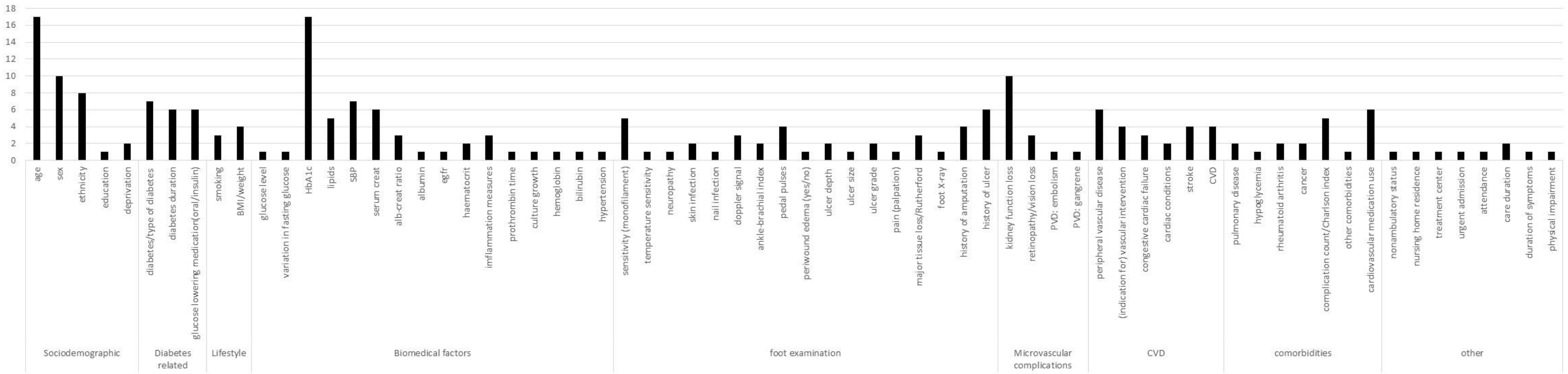

ESM Figure 3: summary of the risk of bias assessment of the seven domains of the 21 included studies

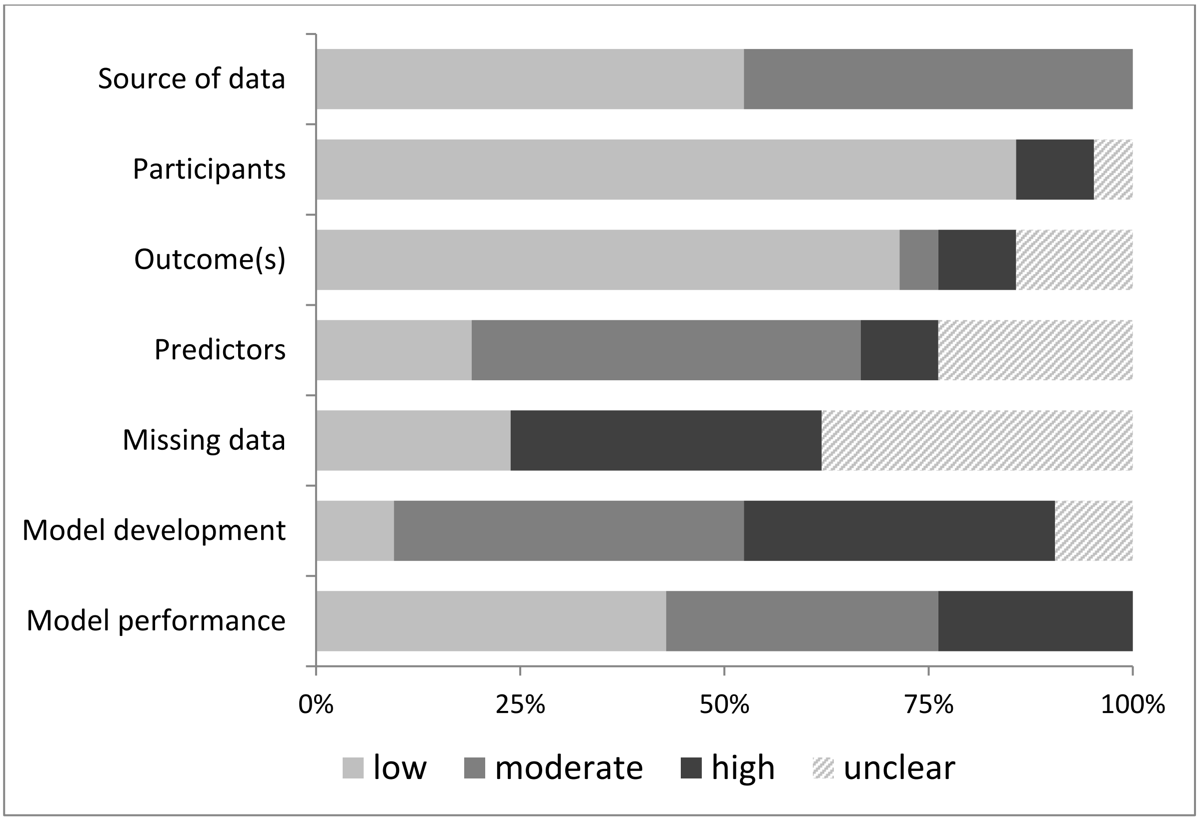

ESM Figure 4: risk of bias assessment of the seven domains of the 21 included studies.

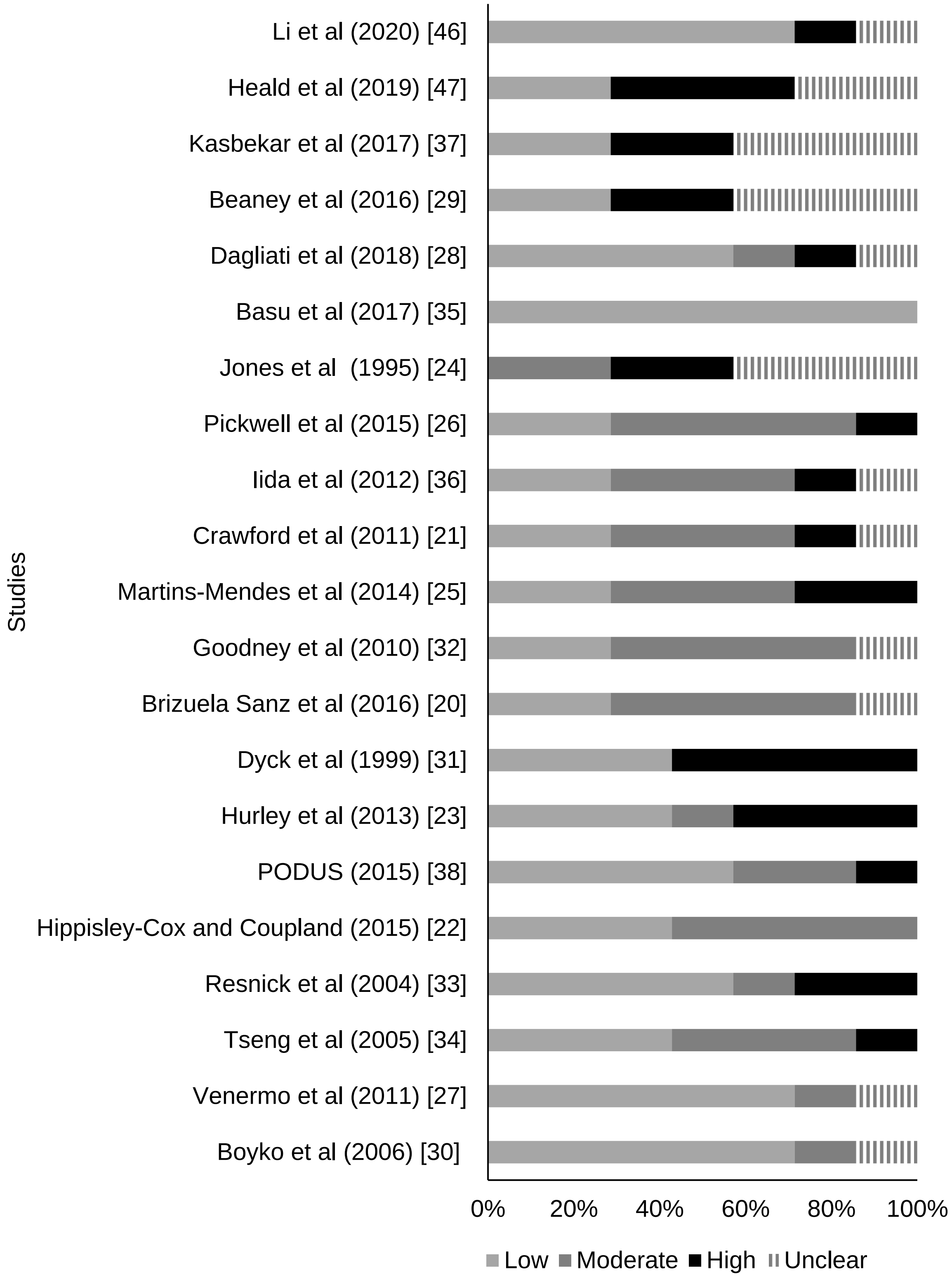

ESM Figure 5. Calibrations plots for four prognostic models predicting amputation before recalibration

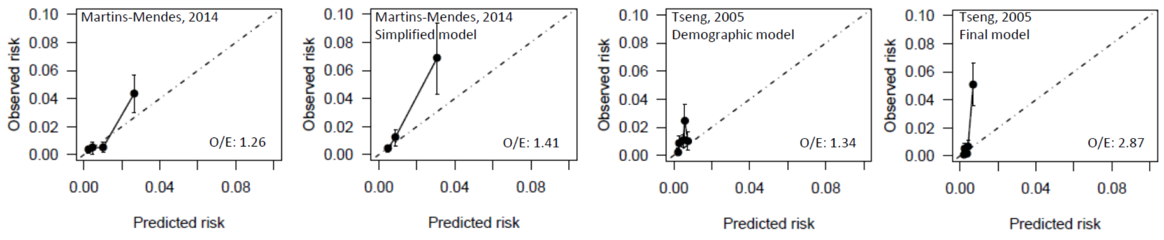

ESM Figure 6: Calibration plot of seven prognostic models for amputation after recalibration

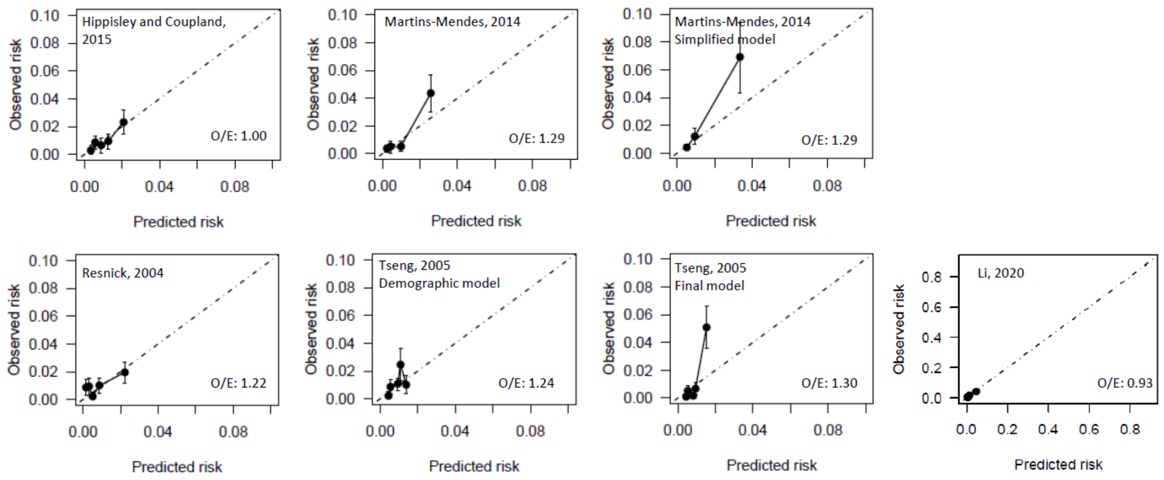

ESM Figure 7: Calibration plots for three prognostic models for foot ulcer before recalibration

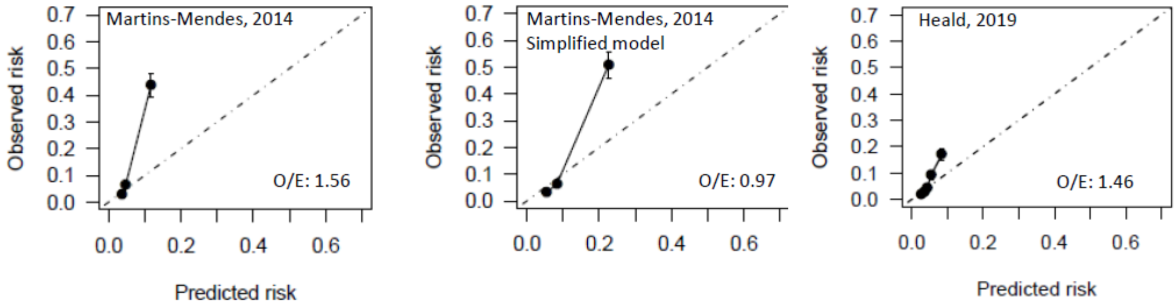

ESM Figure 8: Calibration plots for six prognostic models for foot ulcer after recalibration.

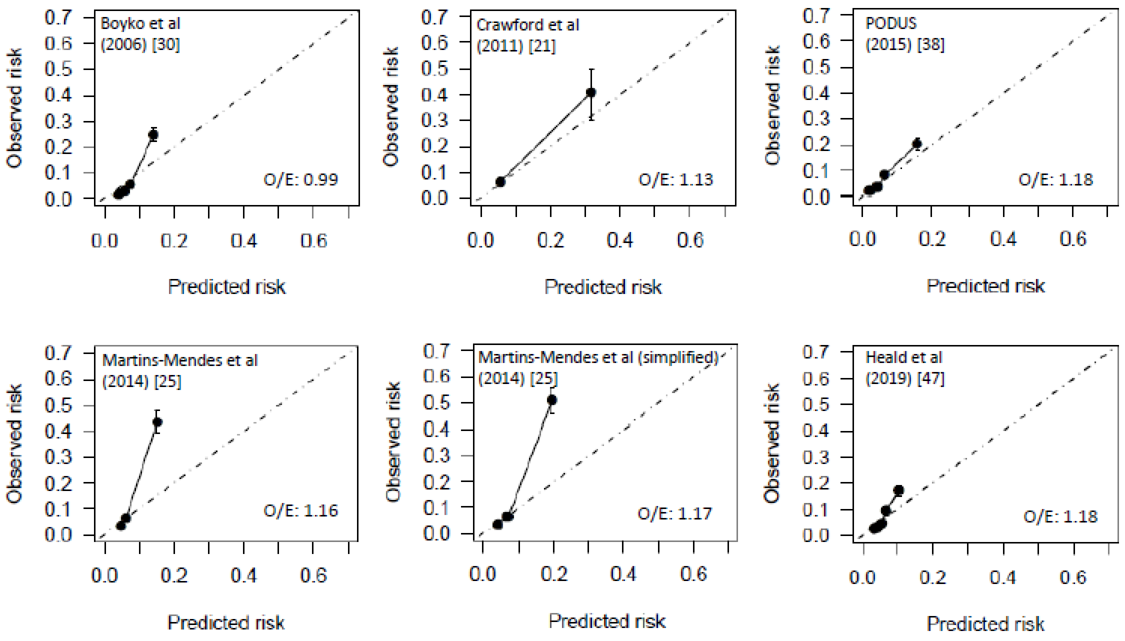

Supplement: Supplementary file 1 — (PDF 736 kb) [file 125_2021_5448_MOESM1_ESM.pdf]
